# Assessment of bacterial and viral gut communities in healthy and tumoral colorectal tissue using RNA and DNA deep sequencing

**DOI:** 10.1002/cam4.6483

**Published:** 2023-08-28

**Authors:** Ainhoa Garcia‐Serrano, Dhananjay Mukhedkar, Emilie Hultin, Ulla Rudsander, Yvonne Wettergren, Agustín Enrique Ure, Joakim Dillner, Laila Sara Arroyo‐Mühr

**Affiliations:** ^1^ Department of Clinical Science, Intervention and Technology (CLINTEC) Karolinska Institutet Stockholm Sweden; ^2^ Hopsworks AB Stockholm Sweden; ^3^ Department of Surgery Sahlgrenska University Hospital, Sahlgrenska Academy at University of Gothenburg Gothenburg Sweden; ^4^ Center for Cervical Cancer Elimination Forskningsgatan F56 Karolinska University Hospital Huddinge Stockholm Sweden

**Keywords:** colorectal cancer, deep‐sequencing, gut microbiome

## Abstract

**Background:**

Colorectal cancer (CRC) is known to present a distinct microbiome profile compared to healthy mucosa. Non‐targeted deep‐sequencing strategies enable nowadays full microbiome characterization up to species level.

**Aim:**

We aimed to analyze both bacterial and viral communities in CRC using these strategies.

**Materials & Methods:**

We analyzed bacterial and viral communities using both DNA and RNA deep‐sequencing (Novaseq) in colorectal tissue specimens from 10 CRC patients and 10 matched control patients. Following taxonomy classification using Kraken 2, different metrics for alpha and beta diversities as well as relative and differential abundance were calculated to compare tumoral and healthy samples.

**Results:**

No viral differences were identified between tissue types, but bacterial species *Polynucleobacter necessarius* had a highly increased presence for DNA in tumors (*p* = 0.001). RNA analyses showed that bacterial species *Arabia massiliensis* had a highly decreased transcription in tumors (*p* = 0.002) while *Fusobacterium nucleatum* transcription was highly increased in tumors (*p* = 0.002).

**Discussion:**

Sequencing of both DNA and RNA enables a wider perspective of micriobiome profiles. Lack of RNA transcription (Polynucleobacter necessarius) casts doubt on possible role of a microorganism in CRC. The association of F. nucleatum mainly with transcription, may provide further insights on its role in CRC.

**Conclusion:**

Joint assessment of the metagenome (DNA) and the metatranscriptome (RNA) at the species level provided a huge coverage for both bacteria and virus and identifies differential specific bacterial species as tumor associated.

## 1 INTRODUCTION

Human microbiome studies have grown exponentially in the last decades, documenting several key associations to health and disease. Changes in microbiome diversity and dysbiotic states have been related to several diseases such as diabetes, obesity, autoimmune diseases, and cancer.^1^, ^2^, ^3^, ^4^, ^5^ The Hallmarks of Cancer now includes polymorphic microbiomes as new enabling characteristics, highlighting its importance in both the carcinogenic processes as well as in prognosis or development of resistance to chemotherapy.^6^

Colorectal cancer (CRC) is the third most common incident cancer worldwide and the second in terms of mortality.^7^ CRC is one of the most studied cancers when it comes to the human microbiome.^8^ Diet is a major CRC risk factor and the microbiome has a direct effect on the nutrient metabolism in the colorectal epithelium.^1^, ^3^, ^9^ The ecological composition of colonic mucosa can directly influence tissue microenvironment and its functionality not only by the presence of certain microorganisms but also by their metabolic outputs.^9^ It has been widely demonstrated how gut microbiome shifts can lead to proinflammatory states that favors tumorigenic processes.^6^, ^10^ Many bacterial taxa such as enterotoxigenic *Bacteroidetes fragilis* (ETBF) or *Escherichia coli* pks + have been related with tissue damage and DNA mutations via the production and secretion of bacterial enterotoxins.^11^ Even so, the most CRC‐associated bacterium is *Fusobacterium nucleatum* spp.^12^, ^13^, ^14^, ^15^ The abundance of *F. nucleatum* has been strongly associated not only with the presence of CRC but also with patient outcomes and even with chemotherapy resistance.^12^, ^13^, ^14^, ^15^

Although many studies have described the human gut microbiome,^16^, ^17^ microbiome assessment techniques vary greatly both in the laboratory phase and in the bioinformatic analysis phase. As next generation sequencing (NGS) technologies are nowadays widely available at low costs, the power of this technology should be used to increase the resolution of the microbiome profile in CRC. In previous work with part of the samples used in the present study, we investigated the differences between healthy mucosa and tumor tissue from CRC patients as well as the differences between those CRC patients and healthy subjects using 16S rRNA sequencing.^18^ These analysis focused only on bacterial communities and were restricted most of the times to a genus level classification due to the technical limitations of only sequencing the 16S rRNA gene.^19^ In the present study, these issues were addressed by studying both bacterial and viral communities at the species level in colorectal human samples using both metagenomics and metatranscriptomics (both DNA and RNA deep‐sequencing).

## 2 METHODS

### 2.1 Clinical study protocol

The present study includes biopsies from 10 patients who were diagnosed with Stages I–III colon cancer after colonoscopy examination and 10 patients that acted as controls where no tumor (neither malignant nor benign) was seen during colonoscopy examination. Cases and controls were matched by age and sex and description of all tumors and 2/10 controls as well as results on their 16S rRNA sequencing analysis have previously been published.^18^ Characteristics of age, sex, body mass index (BMI) and tumor stage from these 20 patients can be seen in Table 1. The study was approved by the Regional Ethical Review Board in Gothenburg under study number 233‐10 and registered at ClinicalTrials.gov (ID: NCT03072641). Informed consent was obtained from all study subjects. All research was performed in accordance with relevant guidelines and regulations.

**TABLE 1:** Clinical information for patients included in the study.

Group	CRC patients	Non‐CRC subjects
Sample size	*n* = 10	*n* = 10
Sex	7 Females	7 Females
	3 Males	3 Males
Age		
Mean(SD)	71.5 (9.78)	70.8 (9.77)
BMI		
Mean(SD)	24.96 (3.28)	NA
Stage	I (1), II (4), III (5)	‐

Abbreviations: CRC, colorectal cancers; SD, standard deviation.

### 2.2 DNA and RNA extraction

DNA was extracted using the AllPrep DNA/RNA/Protein Kit (Qiagen), followed by polymerase chain reaction (PCR) inhibitor removal with the OneStep‐96 PCR Inhibitor Removal Kit (Zymo Research), and DNA concentration measurement with the Qubit 2.0 Fluorometer (Life Technologies). Total RNA was extracted with the AllPrep DNA/RNA/Protein Kit (Qiagen) and reverse transcribed to single‐stranded cDNA using the high‐capacity cDNA reverse transcription kit (ThermoFisher Scientific) following manufacturer's instructions. Double‐stranded cDNA was prepared following step 2 of the Maxima H Minus Double‐Stranded cDNA Kit (ThermoFisher Scientific) and the cDNA was cleaned using the GeneJET PCR Purification Kit (ThermoFisher Scientific). Thereafter, cDNA was subjected to PCR inhibitor removal with an OneStep‐96 PCR Inhibitor Removal Kit (Zymo Research) and cDNA concentration measurement with a Qubit 2.0 Fluorometer (Life Technologies). Both DNA and cDNA were stored at −20°C until further analysis.

### 2.3 Whole genome and whole transcriptome sequencing

Extracted material (DNA and cDNA) was thereafter subjected to library preparation, using the Nextera XT DNA library preparation kit (Illumina) following the manufacturers' reference guide, starting with 1 ng of DNA/cDNA (as recommended by the manufacturer) and using unique indexed adapters to facilitate pooling of the libraries. Libraries were individually quantified using the QuantiFluor system (Promega) and the library sizes were measured using the Bioanalyzer (Agilent) as quality analysis. All 20 libraries were normalized to 2 nM and pooled prior paired‐end sequencing using NovaSeq 6000 system (Illumina) at 2 × 150 bp aiming for 100 M high‐quality paired end reads/sample.

### 2.4 Bioinformatic preprocessing

Indexes, included in the Illumina adapters, were used to assign raw sequence reads obtained from the NextSeq500 (Illumina) platform to the originating samples. Reads were quality checked and adapter trimmed with Trimmomatic using 36 bp as minimal length for the reads.^20^ High‐quality reads were screened against the human reference genome hg19 using NextGenMap^21^ and only reads that did not map to the human genome, with >95% identity over 75% of their length, were considered as non‐human and further analyzed for microbiome. Once human reads were filtered from the data set, high‐quality non‐human reads were classified using Kraken2 v. 2.1.1,^22^ which was run against a reference database containing all RefSeq bacterial and viral genomes (built in December 2020) with a 0.1 confidence threshold.

### 2.5 Diversity analysis and statistics

All diversity analyses were performed at the species level using R (v.4.2.2). Packages used in this analysis were biomformat,^23^ phyloseq,^24^ ggvenn,^25^ tidyr,^26^ ggpubr,^27^ vegan,^28^ vtable,^29^ metagenomeSeq,^30^ funrar,^31^ and superheat.^32^ The biom files generated with kraken‐biom^33^ were used, together with sample metadata, to construct phyloseq objects. Results reported all species which comprised more than 0.1% (in at least one sample) of total bacterial or viral reads, separately. Bacterial and viral species relative abundance comparison using group *F*‐tests were carried out for healthy mucosa and tumors in both DNA and RNA datasets (Tables S1–S4). Relative abundance plots for top 10 species were plotted for tumor and healthy mucosa in both DNA and RNA datasets. Observed species, Shannon and inverse Simpson alpha diversity indexes were calculated after rarefaction (between 68,535 and 5,034,325 reads, depending on the subset analyzed) to standardize species representation regardless of sample depth. Differences between groups for alpha diversity were calculated using unpaired Wilcoxon tests. Bray–Curtis and Jaccard beta diversity indexes were calculated to analyze differences between bacterial and viral communities, visualized using principal component analysis (PCoA) and ANOSIM tests were used to stablish if differences between groups were greater than differences within groups. Finally, differential abundance (DA) analysis comparing tumor and healthy tissue was performed for DNA and RNA separately. In this last step, species counts were transformed using cumulative sum scaling (CSS), log2 transformation, as well as a pseudocount addition to handle data sparsity. *P*‐values obtained from DA analysis were corrected using False Discovery Rate (FDR). Statistical significance was obtained when *p* < 0.01.

## 3 RESULTS

### 3.1 Sequencing coverage

Raw input fastq files including both human and non‐human reads had an average size of 18.24GB per sample (minimum 12.5GB and maximum 21.2GB) for DNA and an average size of 10.54GB per sample (minimum 1.3GB and maximum 19.4GB) for RNA. Human reads constituted 83.73% (minimum 42% and maximum 88.38%) of the reads per sample on average for DNA and 46.25% of the reads per sample on average (29.76% minimum and 62.55% maximum) for RNA. After removing human reads, an average depth of 17,691,220 reads per sample were remaining (minimum 8,035,413 and maximum 23,419,122) for DNA and an average coverage of 47,073,844 reads per sample (1,228,129 minimum and 121,381,065 maximum) for RNA.

### 3.2 Taxonomic classification

Bacterial taxonomic resolution at the species level was at 89.94% for DNA sequencing reads and 88.36% for RNA sequencing reads. We identified up to 5918 species corresponding to 6580 taxa when performing metagenomics and 5915 species within 6694 taxa when analyzing metatranscriptomics. For viral species, taxonomic resolution was slightly higher, reaching 90.23% for DNA sequencing reads and 89.68% for RNA sequencing reads (628 species were identified from 696 taxa when performing metagenomics and 391 species from 436 taxa when performing metatranscriptomics).

**FIGURE 1:**
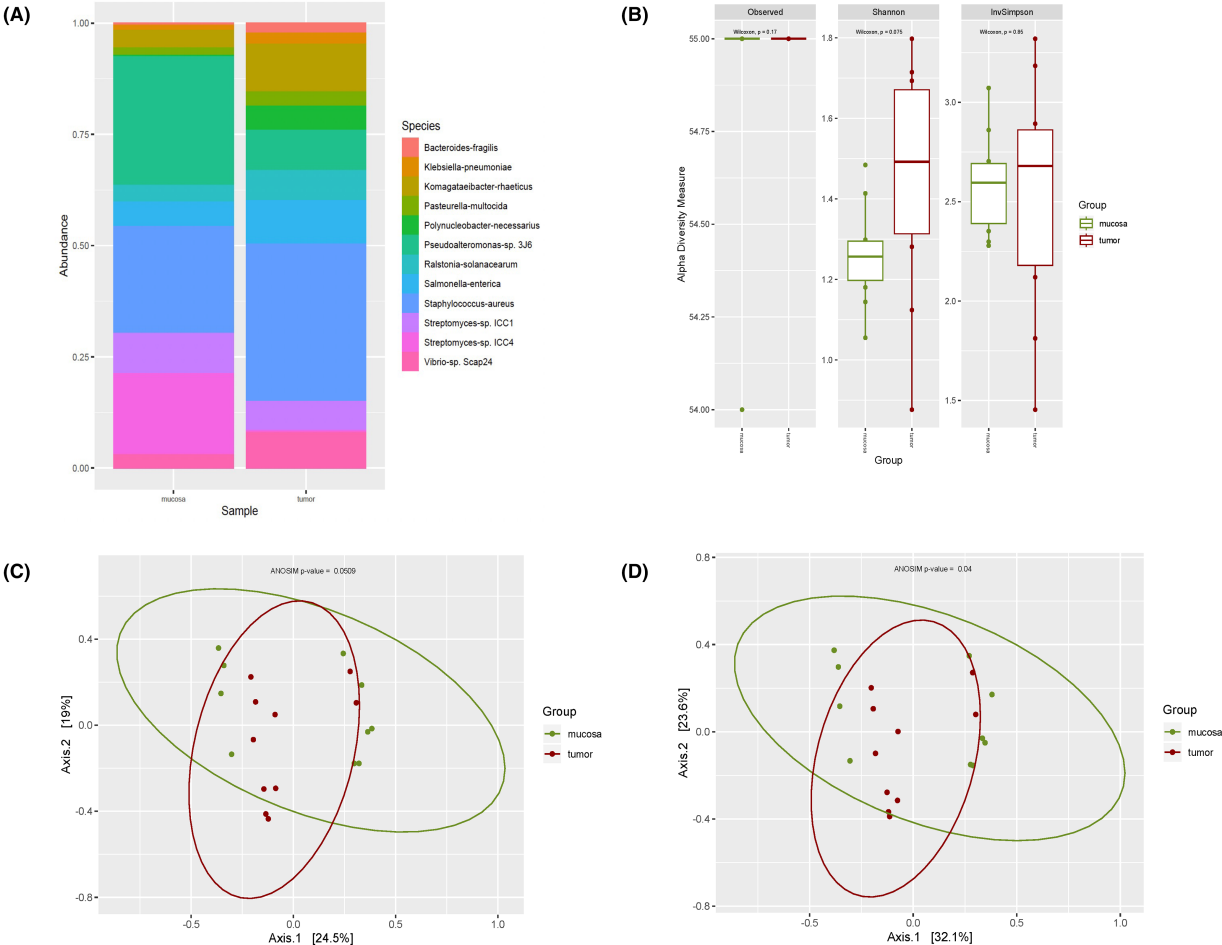
DNA Bacterial diversity of filtered reads. (A) Relative abundance grouped for top 10 bacterial species per group. (B) Observed, Shannon and Inverse Simpson alpha diversity indexes rarefacted at 5M reads. (C) Jaccard beta diversity PCoA. (D) Bray–Curtis beta diversity PCoA.

**FIGURE 2:**
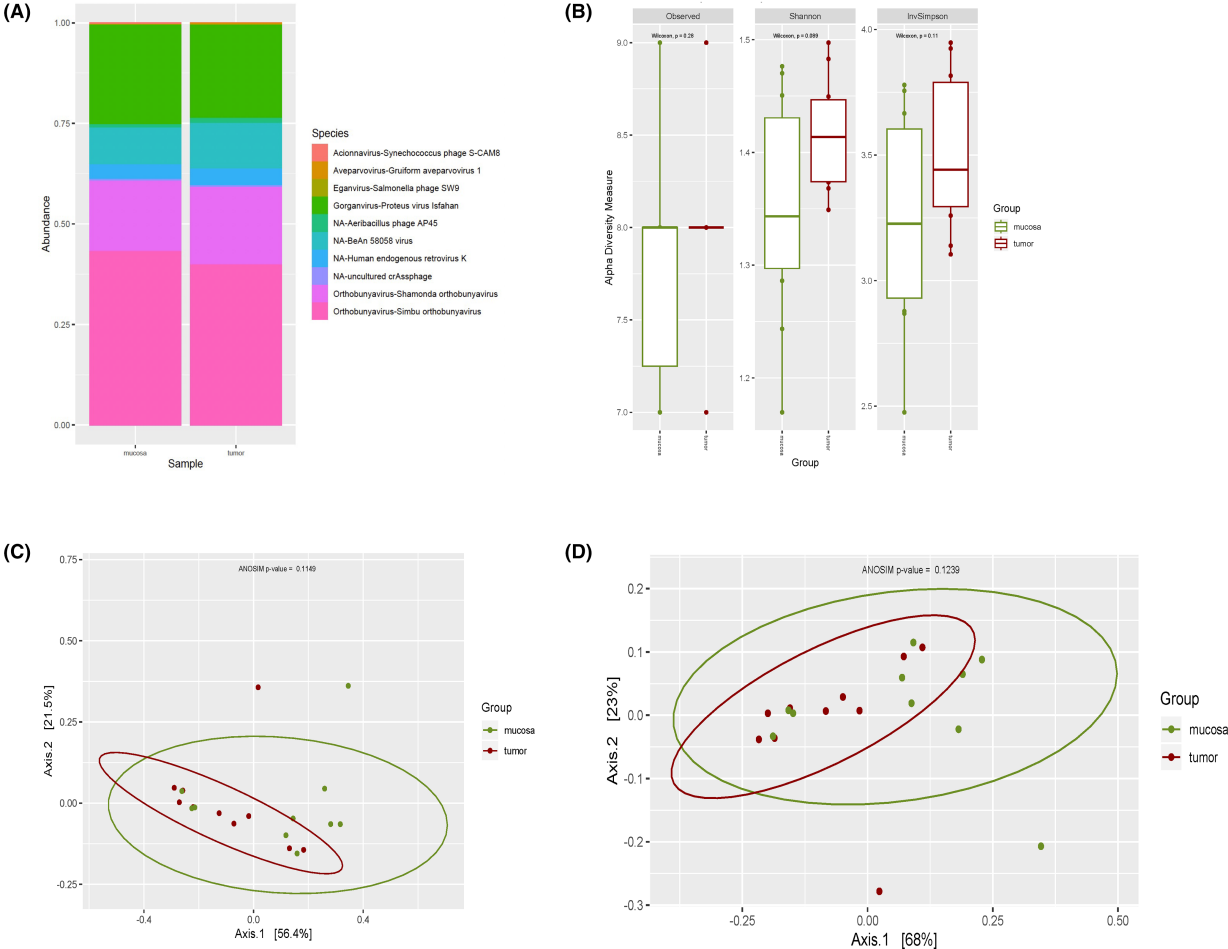
DNA Viral diversity of filtered reads. (A) Relative abundance grouped for top 10 virus species per group. (B) Observed, Shannon and Inverse Simpson alpha diversity indexes rarefacted at 68K. (C) Jaccard beta diversity PCoA. (D) Bray–Curtis beta diversity PCoA.

Filtering by a minimum of 0.1% of relative abundance in at least one sample translated into detection of 55 and 64 bacterial species for DNA and RNA sequencing analysis, respectively. The top 10 bacterial species per group are presented in Figures 1A (DNA) and 3A (RNA), and the relative abundances of the complete list of filtered species are available in Tables S1 (DNA) and S3 (RNA). Ten viral species were detected by metagenomics and six viral species were identified using metatranscriptomics, after filtering. Viral species relative abundances are presented in Figures 2A (DNA), 4A (RNA) and in Tables S2 (DNA) and S4 (RNA).

### 3.3 Tumor versus healthy mucosa (DNA)

#### 3.3.1 Bacteria

Comparing the bacterial species between colorectal tumor specimens and healthy mucosa by analyzing the relative abundance revealed statistically significant differences (*p* < 0.01) for 12 bacterial species (Table S1). Among those, *Polynucleobacter necessarius* (reported not only to be found mainly in water ponds, but also to be significantly enriched in patients with septic shock and as an important antibiotic‐resistant gene host^34^, ^35^, ^36^), was the species with highest *F*‐value, being more abundant in tumors (Figure 1A, Table S1; *F* = 24.652, *p* < 0.01).

Alpha diversity indexes, which informed about diversity within samples, did not show any statistical differences between groups (Figure 1B). Beta indexes, which informed about differences in bacterial communities diversity among tumors and healthy tissue, were assessed with two different indexes (Figure 1C,D), Jaccard and Bray–Curtis indexes, which showed a clear clustering but not reaching statistically significance. DA analysis revealed only one bacterial species being more abundant in tumors when comparing with healthy tissue: *Polynucleobacter necessarius* (Table 2; log2 fold change of 6.5 and adjusted *p* = 0.001).

**TABLE 2:** Differentially (*p* < 0.01) abundant bacteria (healthy mucosa vs tumor) in DNA and RNA datasets.

Group	Species	Log2 fold change	adj *p*‐value
DNA	*Polynucleobacter necessarius*	6.5	0.001
RNA	*Arabia massiliensis*	−12.68	0.0001
	*Fusobacterium nucleatum*	5.31	0.002

#### 3.3.2 Virus

Up to 10 viral species were identified when performing metagenomic analysis (Table S2). However, there was no statistically significance found when analyzing relative abundance of species, alpha diversity, beta diversity, or DA analysis (Figure 2).

### 3.4 Tumor versus healthy mucosa (RNA)

#### 3.4.1 Bacteria

**FIGURE 3:**
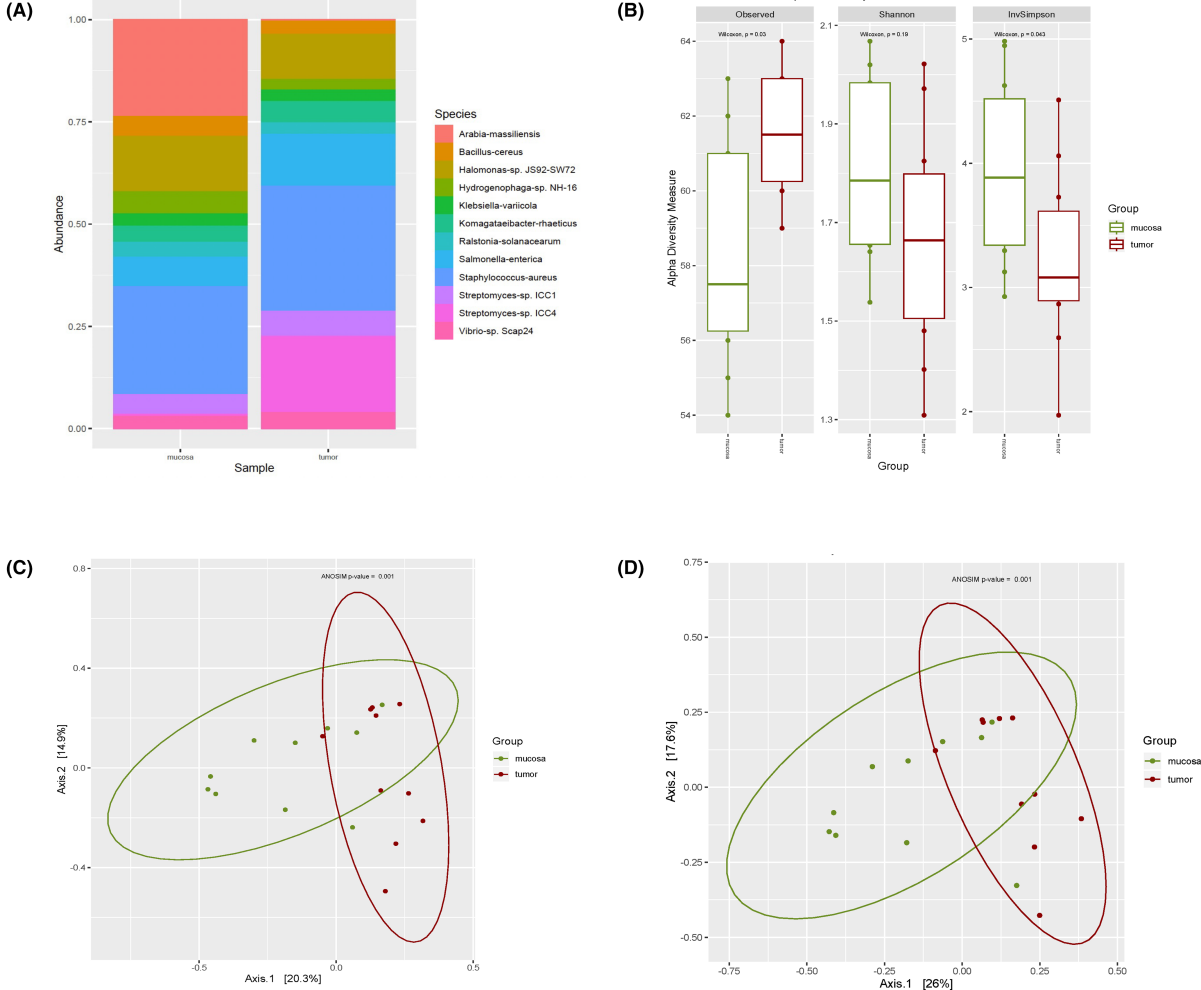
RNA Bacterial diversity of filtered reads. (A) Relative abundance grouped for top 10 bacterial species per group. (B) Observed, Shannon and Inverse Simpson alpha diversity indexes rarefacted at 771K reads. (C) Jaccard beta diversity PCoA. (D) Bray–Curtis beta diversity PCoA.

Comparison of colorectal tumor and healthy mucosa tissue revealed a decreased relative abundance of *Arabia massiliensis* (*F* = 26.021, *p* < 0.01) in tumors (Figure 3A, Table S3). Alpha diversity indexes, which informed about diversity within samples, did not show any statistical differences between groups (Figure 3B). Bacterial communities showed a clear clustering when evaluating Jaccard (*p*_=_ 0.001) and Bray–Curtis (*p* = 0.001) beta diversity indexes (Figure 3C,D). DA analysis reported two bacterial species: *Arabia massiliensis* being more abundant in healthy tissue (log2 fold change of −12.68 and adjusted *p* = 0.0001) and *Fusobacterium nucleatum* (log2 fold change of 5.31 and adjusted *p* = 0.002) being more abundant in tumors (Table 2).

#### 3.4.2 Virus

**FIGURE 4:**
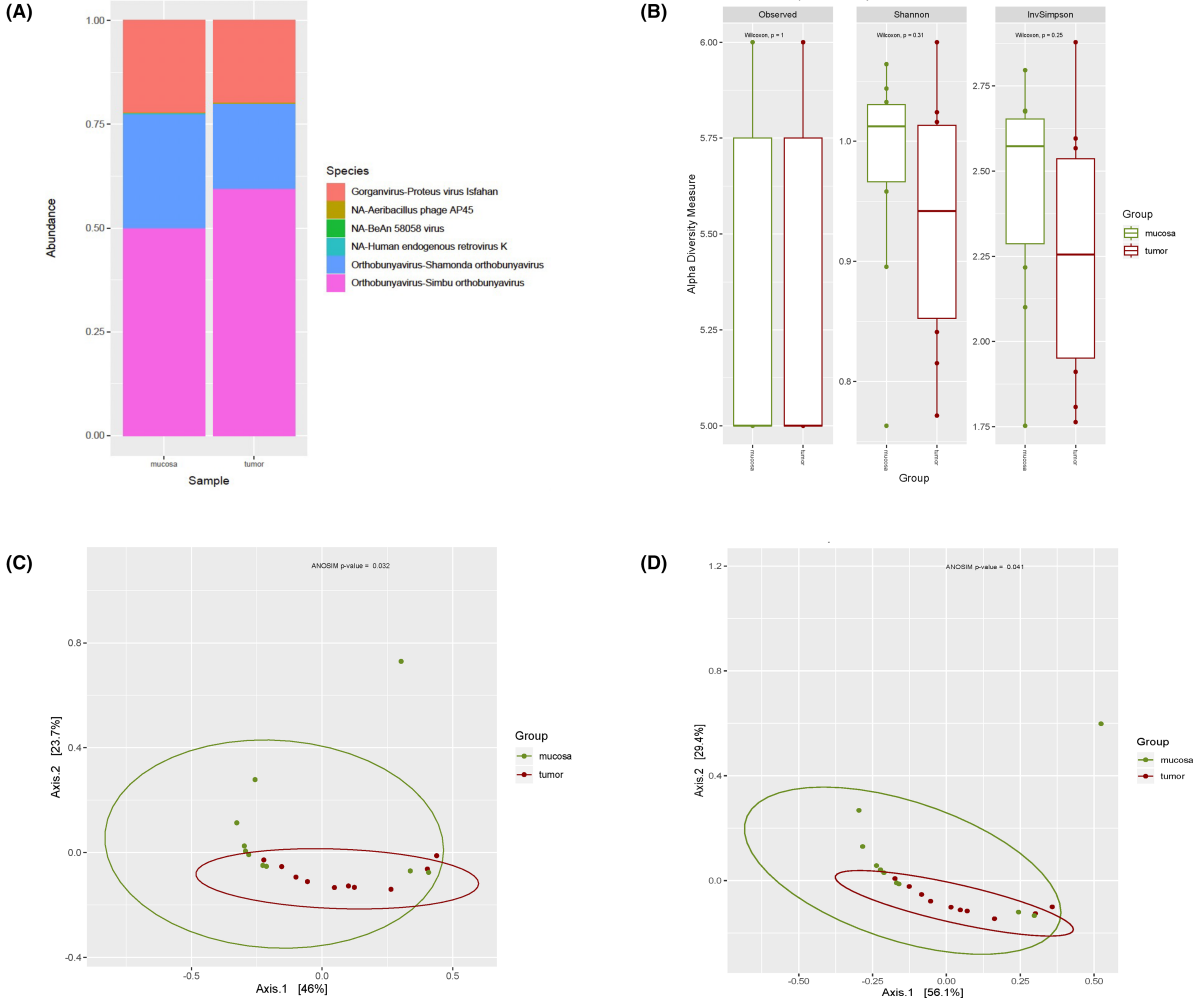
RNA Viral diversity of filtered reads. (A) Relative abundance grouped for top virus species per group. (B) Observed, Shannon and Inverse Simpson alpha diversity indexes rarefacted at 416K. (C) Jaccard beta diversity PCoA. (D) Bray–Curtis beta diversity PCoA.

Up to six viral species were detected when analyzing metatranscriptomes for both tumors and mucosa (Table S4). However, there was no statistically significance found when analyzing species relative abundance, alpha diversity, beta diversity nor DA analysis (Figure 4).

## 4 DISCUSSION

We report an unbiased and deep sequencing of the metagenome and metatranscriptome in CRC cases with non‐CRC controls, identifying three specific bacterial species associated with the disease. *P. necessarius* and *F. nucleatum* were highly increased in tumor specimens when analyzing metagenomics and metatranscriptomics, respectively. Furthermore, *A. massiliensis* was highly decreased in tumor tissue when analyzing RNA data. *P. necessarius* did not reach the established cut‐off when analyzing RNA sequencing data (present in <0.1% of total bacterial transcripts in at least one sample). This species has been mostly identified in freshwater habitats,^34^ but there are studies where the corresponding genus has been reported to be significantly enriched in patients with septic shock and as one important antibiotic resistance gene host.^35^, ^36^ When encountering environmental microorganisms, it is important to identify if those really come from the tumor/specimen, or if those correspond to deposition, and therefore, we highlight the importance of using negative controls to be able to search for microorganisms that may represent deposition/contamination. Also, both DNA and RNA analyses were performed within the exact same samples, and a systematic identification was not detected. Furthermore, observing a lack of RNA transcription casts doubt on possible role of this microorganism in colorectal tumors. Previous studies on skin have detected several hundreds of human papillomavirus types when analyzing DNA sequences, but most of these viruses were not actively transcribed (apparently representing deposition).^37^

Both *A. massiliensis* (recently identified in stool material)^38^ and *F. nucleatum* (the by far most CRC‐associated bacterium in previous studies) were highly detected among the RNA sequences of the mucosa and the tumors, respectively. Compared to DNA analysis, *A. massiliensis* did not surpass the established cut‐off, and *F. nucleatum* did reach it, but its relative abundance presence was low and not different between tumor and healthy mucosa specimens. The absence or low prevalence of these species may be explained by the higher abundance of other species.

Regarding viruses, there were no viral species detected as statistically significant in any of the groups (tumors vs mucosa) despite the depth of sequencing achieved. A recent study on CRC studied the relevance of the interplay between viruses and bacteria, highlighting the importance of the co‐occurrence of bacteriophages and their influence on the bacterial communities.^39^ The present study identified four different phages: all of them were identified while performing metagenomics (*Acionnavirus Synechococcus phage S‐CAM8* [closely related to *Synechococcus phage s rim8*, known to be enriched in late CRC stages^40^], *Eganvirus Salmonella phage SW9, Aeribacillus phage AP45*, and *uncultured crAssphage*) and only one of them was detected when analyzing metatrascriptomics (*Aeribacillus phage AP45*). While 3/4 phages detected in metagenomics showed an increased relative abundance in CRC, as reported by other authors,^41^, ^42^ the present study did not find any statistical significance.

The strengths of the study include that we compared different analysis (relative abundance, alpha and beta diversities as well as DA) for both DNA and RNA sequencing data, aiming to detect bacteria and viruses up to the species level. Prior to analysis, bacterial and viral species were filtered (0.1% of total bacterial/viral reads the presence in at least one sample) to reduce complexity, noise and technical variability while preserving data integrity and representing main communities.^43^ Choosing the appropriate cutoff for filtering is a key step in microbiome analysis since it can strongly influence the downstream results and result in false positivity/negativity.^43^ We also included multiple metrics for each analysis. Up to three different indexes were used to analyze the alpha diversity, offering a wide perspective of within sample diversity, increasing sensitivity for both richness and evenness.^44^ Beta diversity analyses included non‐phylogenetic indexes accounting for both qualitative (Jaccard) and quantitative (Bray–Curtis) indexes to avoid bias of undersampling meanwhile maintaining sensitivity to rare species^45^ and DA analysis to provide more reliable results for specific species enrichment (more than just comparing relative abundances) since this normalized and transformed taxa can account for data sparsity.^30^

The limitations of the study may include the size of samples analyzed. The total number of different specimens was 20 (10 colorectal tumors and 10 healthy mucosa specimens). However, each specimen was sequenced using both DNA and RNA unbiased approaches, and each library generated (total of 40 libraries) provided a very large amount of data (with an average of 18 M and 47 M high‐quality non‐human sequencing reads in DNA and RNA, respectively) detecting >6000 species. Adding data from different studies present in public repositories to increase sample size was avoided to not introduce biases in selection of patients or differences in the exact analysis methods used. The present study was based on a very careful matching of cases and controls as the most reliable strategy.

This study demonstrated how deep sequencing of both DNA and RNA enables a wider perspective of microbiome profiles: individual microbial features can vary depending on whether the metagenome or metatranscriptome is analyzed. Our combination of both DNA and RNA deep sequencing in the same samples revealed that tumors had more diversity in terms of microorganism presence while healthy tissues were more diverse when considering microorganisms with active transcription, in agreement with previous literature.^46^

While *F. nucleatum* is already known to be a potential CRC biomarker for screening, diagnosis, and prognosis prediction for patient outcomes,^12^, ^13^, ^14^, ^15^ the fact that we found a stronger association with transcription of *F. nucleatum* (than merely the presence of the organism) may provide further insights on the role of this microorganism in CRC. Additionally, the great depth achieved with those sequencing methods provides a significant tool for the study of specific genes or transcripts of interest in microbiome‐host interactions.

The role of the recently identified *A. massiliensis* ^38^ and *P. necessarius* needs to be further investigated to elucidate their implication in human health. The present study comprised strict cut‐offs to focus the analysis only on abundant species (species present only in small amounts may conceivably represent contamination and were therefore not studied); however, validation of these “putative” findings would be desirable and findings may need to be considered carefully.

## 5 CONCLUSION

Combining deep sequencing of both metagenome and metatranscriptome allowed a refined microbiome profile of CRC. Diversity differences were stable between DNA and RNA approaches, but the specific tumor‐associated bacteria had some difference depending on whether the metagenome or metatranscriptome was analyzed.

## AUTHOR CONTRIBUTIONS

**Ainhoa Garcia‐Serrano:** Data curation (equal); formal analysis (equal); investigation (equal); methodology (equal); software (equal); writing – original draft (lead); writing – review and editing (equal). **Dhananjay Mukhedkar:** Formal analysis (equal); methodology (equal); software (equal); validation (equal); writing – review and editing (equal). **Emilie Hultin:** Investigation (equal); methodology (lead); writing – review and editing (equal). **Ulla Rudsander:** Conceptualization (equal); project administration (equal); resources (equal); writing – review and editing (equal). **Yvonne Wettergren:** Conceptualization (equal); funding acquisition (equal); resources (lead); supervision (supporting); writing – review and editing (equal). **Agustín Enrique Ure:** Formal analysis (supporting); software (supporting); writing – review and editing (equal). **Joakim Dillner:** Conceptualization (lead); funding acquisition (equal); project administration (lead); resources (equal); supervision (lead); writing – review and editing (equal). **Laila Sara Arroyo‐Mühr:** Data curation (supporting); formal analysis (lead); investigation (equal); methodology (equal); software (supporting); supervision (lead); validation (equal); visualization (equal); writing – original draft (supporting); writing – review and editing (equal).

## FUNDING INFORMATION

This Project was funded by the Nordic Information for Action eScience Center (NIASC), a Nordic Center of Excellence in eScience funded by NordForsk (Project no. 62721), the Swedish state under the LUA‐ALF agreement (grant number ALFGBG‐542821) and by the Human Exposome Assessment Platform (Project No. 874662) granted by Horizon 2020.

## CONFLICT OF INTEREST STATEMENT

The authors declare no competing interests.

## ETHICS STATEMENT

The study was approved by the Regional Ethical Review Board in Gothenburg under study number 233‐10. Informed consent was obtained from all study subjects.

## Supporting information


Table S1.
Table S2.
Table S3.
Table S4.

## Data Availability

Sequencing files for all the aligned, non‐human sequences in both metagenomic and metatranscriptomic data used in this study are available at the Sequence Read Archive (SRA) within the bio‐project ID PRJNA943491(https://www.ncbi.nlm.nih.gov/bioproject/943491).

## References

[1] Valdes AM , Walter J , Segal E , Spector TD . Role of the gut microbiota in nutrition and health. BMJ. 2018;361:k2179. doi:10.1136/bmj.k2179 29899036 PMC6000740

[2] Gomes AC , Hoffmann C , Mota JF . The human gut microbiota: metabolism and perspective in obesity. Gut Microbes. 2018;9(4):308‐325. doi:10.1080/19490976.2018.1465157 29667480 PMC6219651

[3] Dabke K , Hendrick G , Devkota S . The gut microbiome and metabolic syndrome. J Clin Investig. 2019;129(10):4050‐4057. doi:10.1172/JCI129194 31573550 PMC6763239

[4] Miyauchi E , Shimokawa C , Steimle A , Desai MS , Ohno H . The impact of the gut microbiome on extra‐intestinal autoimmune diseases. Nat Rev Immunol. 2023;23(1):9‐23. doi:10.1038/s41577-022-00727-y 35534624

[5] Knippel RJ , Drewes JL , Sears CL . The cancer microbiome: recent highlights and knowledge gaps. Cancer Discov. 2021;11(10):2378‐2395. doi:10.1158/2159-8290.cd-21-0324 34400408 PMC8487941

[6] Hanahan D . Hallmarks of cancer: new dimensions. Cancer Discov. 2022;12(1):31‐46. doi:10.1158/2159-8290.CD-21-1059 35022204

[7] Sung H , Ferlay J , Siegel RL , et al. Global cancer statistics 2020: GLOBOCAN estimates of incidence and mortality worldwide for 36 cancers in 185 countries. CA Cancer J Clin. 2021;71(3):209‐249. doi:10.3322/caac.21660 33538338

[8] Lloyd‐Price J , Abu‐Ali G , Huttenhower C . The healthy human microbiome. Genome Med. 2016;8(1):51. doi:10.1186/s13073-016-0307-y 27122046 PMC4848870

[9] Han S , Zhuang J , Wu Y , Wu W , Yang X . Progress in research on colorectal cancer‐related microorganisms and metabolites. Cancer Manag Res. 2020;12:8703‐8720. doi:10.2147/CMAR.S268943 33061569 PMC7518784

[10] Hale VL , Chen J , Johnson S , et al. Shifts in the fecal microbiota associated with adenomatous polyps. Cancer Epidemiol Biomarkers Prev. 2017;26(1):85‐94. doi:10.1158/1055-9965.EPI-16-0337 27672054 PMC5225053

[11] Tjalsma H , Boleij A , Marchesi JR , Dutilh BE . A bacterial driver‐passenger model for colorectal cancer: beyond the usual suspects. Nat Rev Microbiol. 2012;10(8):575‐582. doi:10.1038/nrmicro2819 22728587

[12] Gethings‐Behncke C , Coleman HG , Jordao HW , et al. Fusobacterium nucleatum in the colorectum, and its association with cancer risk and survival: a systematic review and meta‐analysis. Cancer Epidemiol Biomarkers Prev. 2020;29(3):539‐548. doi:10.1158/1055-9965.EPI-18-1295 31915144

[13] Amitay EL , Werner S , Vital M , et al. Fusobacterium and colorectal cancer: causal factor or passenger? Results from a large colorectal cancer screening study. Carcinogenesis. 2017;38(8):781‐788. doi:10.1093/carcin/bgx053 28582482

[14] Yu TC , Guo F , Yu Y , et al. Fusobacterium nucleatum promotes chemoresistance to colorectal cancer by modulating autophagy. Cell. 2017;170(3):548‐563.e16. doi:10.1016/j.cell.2017.07.008 28753429 PMC5767127

[15] Zhang S , Yang Y , Weng W , et al. Fusobacterium nucleatum promotes chemoresistance to 5‐fluorouracil by upregulation of BIRC3 expression in colorectal cancer. J Exp Clin Cancer Res. 2019;38(1):14. doi:10.1186/s13046-018-0985-y 30630498 PMC6327560

[16] Integrative HMP (iHMP) Research Network Consortium . The integrative human microbiome project. Nature. 2019;569(7758):641‐648. doi:10.1038/s41586-019-1238-8 31142853 PMC6784865

[17] Ehrlich SD , The MetaHIT Consortium . MetaHIT: the European Union project on metagenomics of the human intestinal tract. Metagenomics of the Human Body. Springer; 2011:307‐316. doi:10.1007/978-1-4419-7089-3_15

[18] Hibberd AA , Lyra A , Ouwehand AC , et al. Intestinal microbiota is altered in patients with colon cancer and modified by probiotic intervention. BMJ Open Gastroenterol. 2017;4(1):e000145. doi:10.1136/bmjgast-2017-000145 PMC5609083 28944067

[19] Brumfield KD , Huq A , Colwell RR , Olds JL , Leddy MB . Microbial resolution of whole genome shotgun and 16S amplicon metagenomic sequencing using publicly available NEON data. PloS One. 2020;15(2):e0228899. doi:10.1371/journal.pone.0228899 32053657 PMC7018008

[20] Bolger AM , Lohse M , Usadel B . Trimmomatic: a flexible trimmer for illumina sequence data. Bioinformatics. 2014;30(15):2114‐2120. doi:10.1093/bioinformatics/btu170 24695404 PMC4103590

[21] Sedlazeck FJ , Rescheneder P , von Haeseler A . NextGenMap: fast and accurate read mapping in highly polymorphic genomes. Bioinformatics. 2013;29(21):2790‐2791. doi:10.1093/bioinformatics/btt468 23975764

[22] Wood DE , Lu J , Langmead B . Improved metagenomic analysis with kraken 2. Genome Biol. 2019;20(1):257. doi:10.1186/s13059-019-1891-0 31779668 PMC6883579

[23] McMurdie PJ , Paulson JN . biomformat: an interface package for the BIOM file format. 2022.

[24] McMurdie PJ , Holmes S . phyloseq: an R package for reproducible interactive analysis and graphics of microbiome census data. PLoS One. 2013;8(4):e61217. doi:10.1371/journal.pone.0061217 23630581 PMC3632530

[25] Yan L . ggvenn: Draw Venn Diagram by “ggplot2”. 2021.

[26] Wickham H , Vaughan DGM . tidyr: Tidy Messy Data. 2023.

[27] Kassambara A . ggpubr: “ggplot2” Based Publication Ready Plots. 2022 https://cran.r‐project.org/web/packages/ggpubr/index.html

[28] Oksanen J , Blanchet FG , Michael Friendly RK , et al. vegan: Community Ecology Package. 2020.

[29] Huntington‐Klein N . vtable: Variable Table for Variable Documentation. 2022.

[30] Paulson JN , Stine OC , Bravo HC , Pop M . Differential abundance analysis for microbial marker‐gene surveys. Nat Methods. 2013;10(12):1200‐1202. doi:10.1038/nmeth.2658 24076764 PMC4010126

[31] Grenié M , Denelle P , Tucker CM , Munoz F , Violle C . funrar: an R package to characterize functional rarity. Divers Distrib. 2017;23(12):1365‐1371. doi:10.1111/ddi.12629

[32] Barter RL , Yu B . Superheat: an R package for creating beautiful and extendable heatmaps for visualizing complex data. J Comput Graph Stat. 2018;27(4):910‐922. doi:10.1080/10618600.2018.1473780 30911216 PMC6430237

[33] Dabdoub SM . kraken‐biom: Enabling interoperative format conversion for Kraken results. 2016.

[34] Hahn MW . Isolation of strains belonging to the cosmopolitan polynucleobacter necessarius cluster from freshwater habitats located in three climatic zones. Appl Environ Microbiol. 2003;69(9):5248‐5254. doi:10.1128/AEM.69.9.5248-5254.2003 12957910 PMC194981

[35] Wang C , Li Q , Tang C , et al. Characterization of the blood and neutrophil‐specific microbiomes and exploration of potential bacterial biomarkers for sepsis in surgical patients. Immun Inflamm Dis. 2021;9(4):1343‐1357. doi:10.1002/iid3.483 34288545 PMC8589375

[36] Bai Y , Ruan X , Xie X , Yan Z . Antibiotic resistome profile based on metagenomics in raw surface drinking water source and the influence of environmental factor: a case study in Huaihe River basin, China. Environ Pollut. 2019;248:438‐447. doi:10.1016/j.envpol.2019.02.057 30826606

[37] Hultin E , Arroyo Mühr LS , Lagheden C , Dillner J . HPV transcription in skin tumors. PLoS One. 2019;14(5):e0217942. doi:10.1371/journal.pone.0217942 31150522 PMC6544312

[38] Traore SI , Azhar EI , Yasir M , et al. Description of ‘Arabia massiliensis’ gen. nov., sp. nov., ‘Gordonibacter massiliensis’ sp. nov., and ‘Bacilliculturomica massiliensis’ gen. Nov., sp. nov., isolated from a faecal specimen of a 50‐year‐old Saudi Bedouin woman. New Microbes New Infect. 2017;19:87‐90. doi:10.1016/j.nmni.2017.05.011 28794883 PMC5537403

[39] Li M , Wang C , Guo Q , et al. More positive or more negative? Metagenomic analysis reveals roles of virome in human disease‐related gut microbiome. Front Cell Infect Microbiol. 2022;12:846063. doi:10.3389/fcimb.2022.846063 35493727 PMC9040671

[40] Nakatsu G , Zhou H , Wu WKK , et al. Alterations in enteric Virome are associated with colorectal cancer and survival outcomes. Gastroenterology. 2018;155(2):529‐541.e5. doi:10.1053/j.gastro.2018.04.018 29689266

[41] Shen S , Huo D , Ma C , Jiang S , Zhang J . Expanding the colorectal cancer biomarkers based on the human gut Phageome. Microbiol Spectr. 2021;9(3):e0009021. doi:10.1128/Spectrum.00090-21 34935421 PMC8693921

[42] Zuo W , Michail S , Sun F . Metagenomic analyses of multiple gut datasets revealed the association of phage signatures in colorectal cancer. Front Cell Infect Microbiol. 2022;12:918010. doi:10.3389/fcimb.2022.918010 35782128 PMC9240273

[43] Cao Q , Sun X , Rajesh K , et al. Effects of rare microbiome taxa filtering on statistical analysis. Front Microbiol. 2021;11:607325. doi:10.3389/fmicb.2020.607325 33510727 PMC7835481

[44] DeJong TM . A comparison of three diversity indices based on their components of richness and evenness. Oikos. 1975;26:222. doi:10.2307/3543712

[45] Beck J , Holloway JD , Schwanghart W . Undersampling and the measurement of beta diversity. Methods Ecol Evol. 2013;4:370‐382. doi:10.1111/2041-210x.12023

[46] Yao Q , Tang M , Zeng L , et al. Potential of fecal microbiota for detection and postoperative surveillance of colorectal cancer. BMC Microbiol. 2021;21(1):156. doi:10.1186/s12866-021-02182-6 34044781 PMC8157663

